# Anesthesia for awake craniotomy: a case report

**DOI:** 10.1093/jscr/rjad521

**Published:** 2023-09-16

**Authors:** Ahmed Khaled Alanzi, Samah Hakmi, Shahid Adeel, Samar Yaser Ghazzal

**Affiliations:** Anesthesia Department, King Hamad University Hospital, Building 2435, Road 2835, Block 228, P.O Box 24343, Busaiteen, Kingdom of Bahrain; Anesthesia Department, King Hamad University Hospital, Building 2435, Road 2835, Block 228, P.O Box 24343, Busaiteen, Kingdom of Bahrain; Anesthesia Department, King Hamad University Hospital, Building 2435, Road 2835, Block 228, P.O Box 24343, Busaiteen, Kingdom of Bahrain; Anesthesia Department, King Hamad University Hospital, Building 2435, Road 2835, Block 228, P.O Box 24343, Busaiteen, Kingdom of Bahrain

**Keywords:** seizures, complication, anesthesia, maintenance, brain

## Abstract

Awake craniotomy (AC) is a neurosurgical technique that enables the precise localization of functional neural networks through intraoperative brain mapping and real-time monitoring. This operative method has been popularized in recent years due to decreased postoperative morbidities. We present a case of 31-year-old female who was presented with episodes of generalized tonic colonic seizures. She had a history of recurring seizures. Upon further investigations, she was diagnosed with brain space-occupying lesions initially suspected as low-grade glioma. Considering the lesion site, the patient was deemed a suitable candidate for an AC. To achieve conscious sedation, the patient received infusions of remifentanil and propofol at varying rates. During the procedure, the patient was under sedation and was regularly tested for response to predetermined commands. The tumor was successfully excised by using a combination of local anesthesia on the scalp and by the administration of propofol and boluses through a systemic infusion.

## Introduction

Awake craniotomy (AC) is an intracranial surgical procedure in which the patient is maintained in a state of partial consciousness [1]. Historically, this procedure has been used for the treatment of seizures and epilepsy. However, it has evolved over the past century to become a widely accepted technique for a range of intracranial conditions, such as primarily gliomas, due to their close proximity with eloquent areas in the brain [2]. AC was first performed under conscious sedation by Wilder Penfield in 1937, who believed that the patient’s consciousness and alertness should be preserved through the use of local anesthesia (LA) [3]. He professed that the patient should be able to describe sensations while maintaining minimal pain. In recent times, AC has also been employed in various conditions, including arteriovenous malformations, supratentorial tumors, and mycotic aneurysms [4]. The purpose of this procedure is to accurately localize and remove lesions while simultaneously assessing the patient’s motor, sensory, and language capabilities in real time. This improves the postoperative functional neurological status and quality of life [5].

## Case presentation

**Figure 1:**
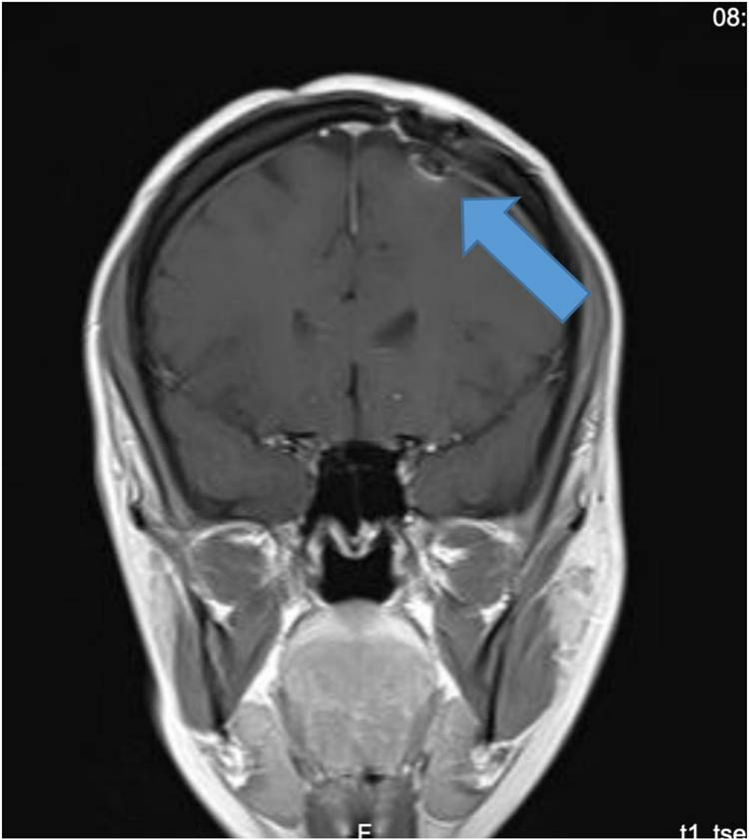
Preoperative CT of the brain.

**Figure 2:**
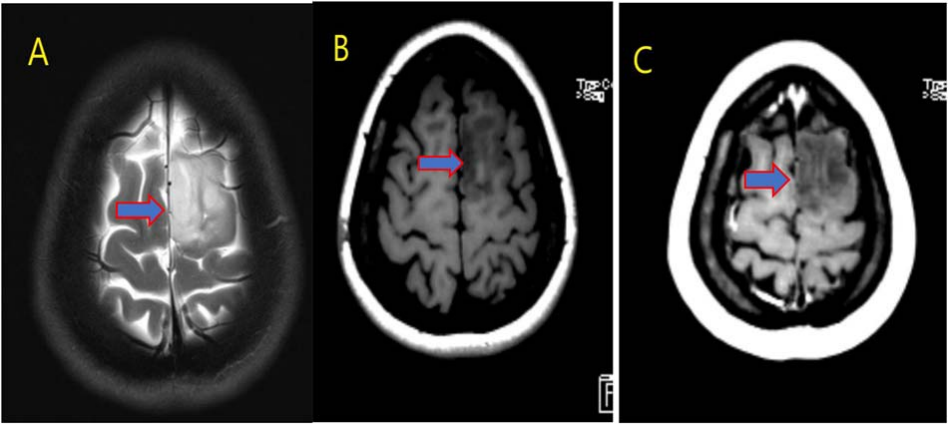
MRI brain with contrast; (A) T2, (B) T1, and (C) T1 post contrast; space occupying cortical mass lesion in the left frontal lobe/superior frontal gyrus showing mild faint minimal contrast enhancement (C) (low-grade glioma).

**Figure 3:**
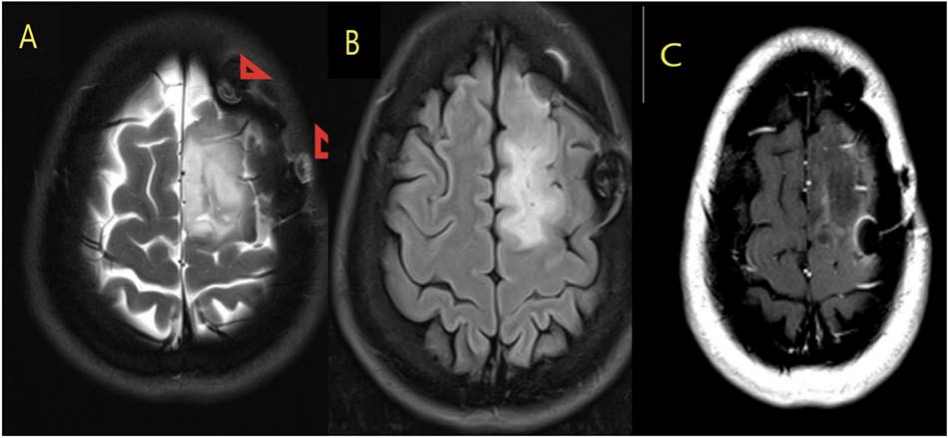
MRI brain with contrast (A) T2, (B) FLAIR, and (C) T1 postcontrast; in comparison to the fore mentioned study; still noted postoperative changes in the form of craniotomy (regression of size of space occupying cortical mass lesion with mild surrounding vasogenic edema in the left frontal lobe/superior frontal gyrus).

Our patient is a 31-year-old female who presented to the emergency department due to two episodes of generalized tonic colonic seizures. Upon presentation, the patient was administered with supplemental oxygen. She was drowsy and confused while exhibiting signs of right-sided hemiparesis and paralysis. Her pupils were 3 mm and reacted briskly while she showed good left-sided localizing. Her seizures terminated after intravenous administration of midazolam at 5 mg. Further, it was known that she has been symptomatic for 1 month and had been suffering from episodes of seizures, with shaking and tremors of upper limbs, and brief episodes of blackouts for about a minute. These episodes were associated with headache and nausea but were self-limited. Seizures recurred two to three times per week. She denied any fall, tongue bite, loss of consciousness, and limb weakness or numbness. A computerized tomography (CT) scan was suggested, which showed left high parietal hypodensity with vasogenic edema (Fig. 1). She was recommended Keppra 1.5 g and dexamethasone 12 mg. Further, a contrast MRI of the brain was performed (Fig. 2). After a thorough evaluation of the patient’s medical records and current condition, it was decided to proceed with an excision of the tumor using the AC technique. To foster trust and comfort, the same anesthesia and surgical teams were assigned to the patient. Before the procedure, the patient was fully informed about the nature of the procedure and the possibility of a failed outcome or further need for a conversion to general anesthesia. To assess the patient’s cognitive and language abilities, preagreed questions and exercises were performed. These included memory-related queries, such as asking for the patient’s phone number and the names of her dog, and motor commands, such as squeezing a squeaky toy. The patient was prepared for awake navigator-assisted left frontal craniotomy for excision of the left frontal lobe space-occupying lesion, which was suspected to be either a low-grade glioma or fibrous dysplasia. During the surgical procedure, a central line was placed in the right jugular vein using ultrasound, followed by the insertion of a right radial arterial line, a urinary catheter, and two additional venous lines. The patient received a continuous flow of 4–6 l of oxygen per minute. To achieve conscious sedation, the patient received infusions of remifentanil and propofol at varying rates. The patient underwent a 5-hour surgical procedure to remove a tumor. During the procedure, the patient was under sedation and was regularly tested for response to predetermined commands. Her conscious sedation level fluctuated, but she never lost consciousness. The removal of the tumor was successful, and postoperative examination revealed normal motor function and cranial nerve function. The patient was taken to the intensive care unit, and postoperative MRI showed a reduction in the size of the mass lesion in the left frontal lobe/superior frontal gyrus along with mild vasogenic edema surrounding it (Fig. 3).

## Discussion

AC has been focused on the identification and excision of any intra-axial lesions that are encroaching upon eloquent cortical areas of the brain that play a crucial role in motor, linguistic, and cognitive functions [6]. Prior to performing AC, it is crucial that a thorough evaluation of absolute and relative contraindications should be considered (Table 1). Communication between all stakeholders involved in the procedure is of paramount importance for the success of the AC procedure [7]. Clear communication with the patient is crucial for a successful procedure, and any impediments that emerge in this relationship can affect the outcome of the procedure [8]. Generally, it includes conditions such as decreased awareness, difficulties with speaking, and confusion. To reduce the risk of confusion during the procedure, preoperative agreements on questions and commands can be made to ensure clear communication [9]. In our case, a different approach was selected, consisting of a combination of LA and conscious sedation, thereby realizing an ‘Awake–Awake–Awake’ technique. Before undergoing the surgical procedure, the administration of LA is performed through scalp blockade, utilizing a combination of bupivacaine and epinephrine for its analgesic properties. The targeted nerve fibers in the scalp region encompass the auriculotemporal, zygomaticotemporal, supraorbital, supratrochlear, and lesser and greater occipital nerves [10]. In our case, conscious sedation was achieved by infusion of remifentanil and propofol at varying rates. The most commonly utilized anesthetic regimen for conscious sedation during surgery involves the systemic administration of propofol in conjunction with short-acting opioids such as fentanyl or remifentanil [11]. This regimen may be administered through continuous infusion, intermittent bolus, or through target-controlled infusion methods. To ensure optimum autonomic respiration, a noninvasive oxygen facial mask is utilized; however, it is crucial to continuously monitor the patient’s respirational frequency and the levels of carbon dioxide expired, as some individuals with inherent susceptibilities may experience obstructive apnea [11].

**Table 1:** Contraindications of AC.

Absolute
Patient refusal
Inability to lay still for any length of time
Inability to cooperate (ex. confusion)
Relative
Patient cough
Learning difficulties
Inability to lay flat
Patient anxiety
Language barriers
Obstructive sleep apnea
Young age

## Conclusion

Our case report illustrates the utilization of a combination of Local/regional anesthesia and sedation as the optimal option for a successful outcome in AC. This case report highlights the importance of the team work among anesthesia and surgeons, where local/regional anesthesia can be combined with sedation to perform a surgical procedure in certain conditions.

## Data Availability

The data that support the findings of this study are available on request from the corresponding author.
